# Biomedical Research and Informatics Living Laboratory for Innovative Advances of New Technologies in Community Mobility Rehabilitation: Protocol for Evaluation and Rehabilitation of Mobility Across Continuums of Care

**DOI:** 10.2196/12506

**Published:** 2022-06-01

**Authors:** Sara Ahmed, Philippe Archambault, Claudine Auger, Audrey Durand, Joyce Fung, Eva Kehayia, Anouk Lamontagne, Annette Majnemer, Sylvie Nadeau, Joelle Pineau, Alain Ptito, Bonnie Swaine

**Affiliations:** 1 School of Physical and Occupational Therapy Faculty of Medicine and Health Sciences McGill University Montreal, QC Canada; 2 Lethbridge-Layton-Mackay Centre for Interdisciplinary Research in Rehabilitation of Greater Montreal Montreal, QC Canada; 3 Center for Outcome Research and Evaluation McGill University Health Center Research Institute Montreal, QC Canada; 4 Centre for Interdisciplinary Research in Rehabilitation of Greater Montreal Montreal, QC Canada; 5 Jewish Rehabilitation Hospital Centre intégré de santé et de services sociaux de Laval Laval, QC Canada; 6 School of Rehabilitation Faculty of Medicine Université de Montréal Montreal, QC Canada; 7 Institut universitaire sur la réadaptation en déficience physique de Montréal Centre intégré universitaire de santé et de services sociaux du Centre-Sud-de-l'Île-de-Montréal Montreal, QC Canada; 8 Computer Science and Software Engineering Department Faculty of Science and Engineering Université Laval Quebec City, QC Canada; 9 School of Computer Science McGill University Montreal, QC Canada; 10 Department of Neurology and Neurosurgery Faculty of Medicine McGill University Health Centre Research Institute Montreal, QC Canada

**Keywords:** health informatics, digital health, individualized care, acquired brain injury, community mobility, participation, physical rehabilitation, virtual reality, artificial intelligence, predictive analytics, biomedical, learning health system

## Abstract

**Background:**

Rapid advances in technologies over the past 10 years have enabled large-scale biomedical and psychosocial rehabilitation research to improve the function and social integration of persons with physical impairments across the lifespan. The Biomedical Research and Informatics Living Laboratory for Innovative Advances of New Technologies (BRILLIANT) in community mobility rehabilitation aims to generate evidence-based research to improve rehabilitation for individuals with acquired brain injury (ABI).

**Objective:**

This study aims to (1) identify the factors limiting or enhancing mobility in real-world community environments (public spaces, including the mall, home, and outdoors) and understand their complex interplay in individuals of all ages with ABI and (2) customize community environment mobility training by identifying, on a continuous basis, the specific rehabilitation strategies and interventions that patient subgroups benefit from most. Here, we present the research and technology plan for the BRILLIANT initiative.

**Methods:**

A cohort of individuals, adults and children, with ABI (N=1500) will be recruited. Patients will be recruited from the acute care and rehabilitation partner centers within 4 health regions (living labs) and followed throughout the continuum of rehabilitation. Participants will also be recruited from the community. Biomedical, clinician-reported, patient-reported, and brain imaging data will be collected. Theme 1 will implement and evaluate the feasibility of collecting data across BRILLIANT living labs and conduct predictive analyses and artificial intelligence (AI) to identify mobility subgroups. Theme 2 will implement, evaluate, and identify community mobility interventions that optimize outcomes for mobility subgroups of patients with ABI.

**Results:**

The biomedical infrastructure and equipment have been established across the living labs, and development of the clinician- and patient-reported outcome digital solutions is underway. Recruitment is expected to begin in May 2022.

**Conclusions:**

The program will develop and deploy a comprehensive clinical and community-based mobility-monitoring system to evaluate the factors that result in poor mobility, and develop personalized mobility interventions that are optimized for specific patient subgroups. Technology solutions will be designed to support clinicians and patients to deliver cost-effective care and the right intervention to the right person at the right time to optimize long-term functional potential and meaningful participation in the community.

**International Registered Report Identifier (IRRID):**

PRR1-10.2196/12506

## Introduction

Acquired brain injury (ABI), including traumatic brain injury (TBI), cerebral palsy (CP)-fetal/perinatal brain injury, and stroke, are the leading causes of disability worldwide [1-3]. According to the World Health Organization, the global incidence of all-severity TBI is estimated at 69 million people, while 15 million people suffer a stroke worldwide each year [4-6]. Statistics Canada indicates that 100,000 Canadians will experience a stroke (59%) or a TBI (71%) each year [6]. Many individuals with mild-to-moderate ABI subsequently return home, yet they continue to experience ongoing cognitive and physical impairments, including mobility limitations resulting in restricted participation in meaningful activities at school, leisure, or work. Worldwide, between 1990 and 2019, there has been an 89% and a 79% increase in individuals with stroke and TBI, respectively, that require rehabilitation services [7]. Individuals with ABI face challenges once discharged from acute care or rehabilitation and with uncertainty regarding their potential for recovery and regaining independence [8]; see also Ahasani et al (unpublished data, March 2021). Mobility limitations in the community are common and affect 30% of persons with TBI [9-11] and up to 50% of stroke survivors [12], even after extensive rehabilitation. Long-term follow-up of individuals with ABI shows that limitations in mobility appear to undergo little change, even 10 years after the initial injury [10,11].

Mobility is a multidimensional construct and consists of the ability to move oneself independently within a “life space,” expanding from one’s home to the neighborhood and beyond [13,14]. The Webber framework identifies 5 vital interrelated determinants that influence mobility (ie, physical, environmental, cognition, psychosocial, and financial) [14] that are also reflected in the International Classification of Functioning, Disability and Health (ICF) framework mobility core set [15]. The built environment also influences community mobility [16,17]. Guided by the Webber and ICF frameworks, studies have shown that diagnosis alone is not enough to predict mobility limitations and that clinical variables (eg, length of hospitalization, performance-based measures), intensity of care, patient-reported outcomes (PROs) of health, and social determinants are needed to accurately predict return-to-work potential, work performance, or social integration [16,17]. In addition, social and healthcare decision makers recognize the importance of modifying features of the social and physical environment to decrease the incidence and severity of disability and enhance mobility and participation [15].

Currently, no reliable measure exists to jointly evaluate the intrinsic and extrinsic factors that influence mobility for individuals with ABI. For the most part, to measure mobility in research, we rely on expensive laboratory technologies and performance-based tools that are burdensome in terms of setup, as well as the time needed from highly qualified professionals, clinicians, or research staff for administration and analysis. Importantly, these tools cannot be readily applied in real-life community contexts. Further, electronic platforms that can collect real-time patient- and clinician-reported data are in their infancy, particularly in rehabilitation.

Rapid advances in technologies over the past 10 years have enabled large-scale biomedical and psychosocial rehabilitation research to improve the function and social integration of persons with physical impairments across the lifespan. In 2017, our team, a network of researchers and clinical partners, established the Biomedical Research and Informatics Living Laboratory for Innovative Advances of New Technologies (BRILLIANT) in community mobility rehabilitation to provide evidenced-based research to improve rehabilitation for individuals with ABI. BRILLIANT builds on the success of the Rehabilitation Living Lab in the Mall (RehabMaLL) [18], a strategic development project and the first transdisciplinary and intersectoral research program to explore the principal (including physical and social) obstacles to and facilitators of participation in public environments for persons with disabilities.

The vision of BRILLIANT is to optimize mobility following ABI across the lifespan. The main objectives are to (1) identify the factors limiting or enhancing mobility in real-world community environments (public spaces, including the mall, home, and outdoors) and understand their complex interplay in individuals of all ages with ABI and (2) customize community environment mobility training by identifying, on a continuous basis, the specific rehabilitation strategies and interventions that patient subgroups benefit from most. Here, we present the research and technology plan for the BRILLIANT initiative.

Working toward the vision of BRILLIANT, the deliverables that emerge from the program will be made available to the broader research and public community and health system partners. The continuously growing BRILLIANT database will provide the wider research community with data to conduct evaluative research, in particular comparative effectiveness research, to identify best practices. Community organizations will also be able to access the data to inform their strategic plans for addressing the needs of their members. The digital solutions will be created with health system partners to ensure they can be scaled up and sustained in the clinical programs. The goal is to create a learning rehabilitation system that allows for continuous garnering of data via technologies that can be shared back with clinicians, patients, and managers to inform clinical decisions.

**Figure 1:**
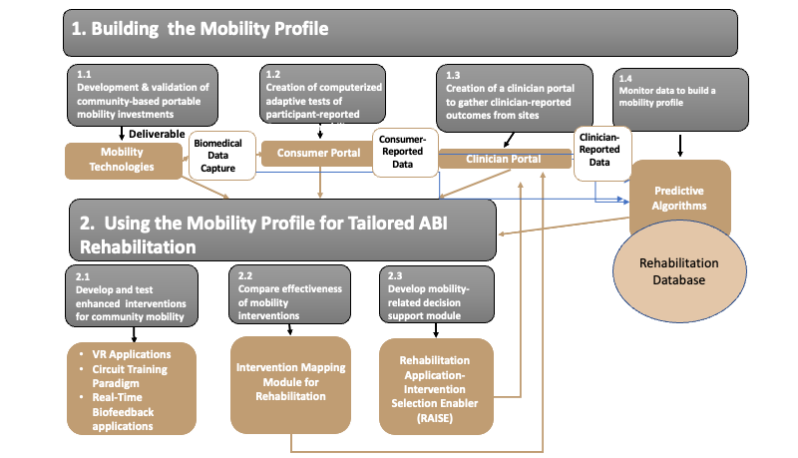
Subcomponents of the Biomedical Research and Informatics Living Laboratory for Innovative Advances of New Technologies (BRILLIANT) to support a learning rehabilitation system. ABI: acquired brain injury; VR: virtual reality. © BRILLIANT.

Here, we present the subcomponents of the BRILLIANT program, referred to as activities that will allow for a multidimensional evaluation of mobility and the factors that influence mobility (Figure 1). Other subcomponents will utilize data generated from the BRILLIANT technologies to evaluate ABI-tailored rehabilitation interventions.

## Methods

### Clinical Sites and Community

A cohort of individuals, adults and children, with ABI (N=1500) will be identified and recruited. These will include patients from the acute care and rehabilitation partner centers within 4 health regions, and the patients will be followed throughout the continuum of rehabilitation services provided by the health care authorities (listed later). Within these 4 health regions, there are 16 clinical programs that provide intensive post-ABI rehabilitation following practice guidelines and in line with other provinces and countries [19-21]. All programs at each center are part of a defined continuum of care for ABI (stroke, TBI, CP). Each of the 4 health regions is responsible for a specific territory covered by the Centre intégré universitaire de santé et de services sociaux (CIUSSS) or the Centre intégré de santé et de services sociaux (CISSS), otherwise known as Quebec health authorities. The 4 sites include the CIUSSS du Centre-Ouest-de-l’Île-de-Montréal (CCOMTL), the CIUSSS Lanaudière, the CISSS de Laval, and the CIUSSS Centre-Sud-de-l’Île-de-Montréal (CCSMTL). Participants will also be recruited from the community to be able to address the BRILLIANT objectives among individuals living with ABI but no longer receiving active rehabilitation.

### Study Population and Recruitment

For those recruited in the clinical settings, the sample will consist of patients and their caregivers, clinicians, coordinators, and managers. A consecutive sample of individuals admitted to acute or rehabilitation care that meet the inclusion criteria will be obtained from admission records. To be eligible to participate, patients or caregivers must be able to (1) provide informed consent to complete questionnaires and provide access to their provincial health data and (2) speak and read English or French.

Within each clinical site, the ABI rehabilitation sites’ managers will obtain a list of emails from clinicians and coordinators interested in participating in the study. A research assistant will obtain consent electronically from clinicians, managers, and patients and caregivers.

Community participants will be recruited through social media and community partner (eg, Brain Injury Canada) organizations. Interested individuals will be invited to call the study coordinator or complete a form on the BRILLIANT website.

### BRILLIANT: Biomedical and Digital Health Technologies and Data Collection

#### Theme I: Building a Mobility Profile

The objective of Theme 1 is to aggregate data from different types of rehabilitation technologies to evaluate the complex interplay between biomedical, personal, and environmental factors that explain variability in mobility. This will be the first time this combination of technologies will be used to generate and validate mobility data that combines brain imaging (BrImagO), biomedical, and patient-reported and clinical measures. This will be accomplished by collecting data among the study population that will be followed through the continuum of care (starting in the hospital) and into the community (up to 2 years post-ABI), enabling us to integrate and analyze their synergistic relationships.

##### Activity 1.1: Development and Validation of Community-Based Portable Mobility Instruments

Moving in community environments is challenging for people with mobility restrictions, as they must adapt to constantly changing contextual/environmental demands (eg, avoid pedestrians/crowds, adapt to noise levels and changing terrains, carry loads). Current laboratory and clinically based evaluations of mobility do not sufficiently capture individuals’ capacity (can do, controlled conditions) and performance (does in real life) as experienced in the community. We will measure complex visually guided mobility tasks (eg, walking and avoiding pedestrians) in community environments, in this case in a shopping mall (Alexis Nihon in Montreal). The mobile technologies we will develop and validate will capture the interaction of patients within the mall environment. Moreover, sensors integrated into assistive technology (eg, wheelchair seating, walkers and canes) will measure the movement, forces, and use of devices. Other sensors, installed in the mall environment, such as the mall floor, stairs, surfaces, and everyday objects, will measure individuals’ movement and the use of key areas in the community environment.

We will also use portable electroencephalography (EEG) to develop metrics that characterize brain activation patterns in key regions of interest (eg, the premotor and motor cortices, posterior parietal cortex) while patients are performing mobility tasks in RehabMaLL. The data collected will provide unprecedented sensitivity that is needed to detect and measure biomedical parameters of mobility in the community. Standardized measurements of biomedical outcomes (BiomedO), taken at various stages of the individuals’ recovery (acute, rehabilitation, and community reintegration phases), will be part of the data used to predict and then classify individuals with ABI into high, medium, or low levels of mobility based on the quality, precision, and coordination of their movement.

At the end of Activity 1.1, we will develop mobility technologies to evaluate personalized longitudinal BiomedO data in a timely manner, a kind of data currently unavailable for individuals with ABI who come to the clinic for follow-up evaluations.

##### Activity 1.2: Patient Outcome Reporting Research System—Creating Computerized Adaptive Tests of Patient-Reported Outcomes for Mobility-Related Domains

PRO data are crucial for understanding mobility as they enable researchers to measure factors influencing mobility that can only be provided by individual participants. The greatest challenge in collecting PROs in the laboratory, clinical, and community settings, however, is the length of time and repetitiveness of standardized paper-based measures, as well as their lack of precision and sensitivity to change [22]. Computerized adaptive tests (CATs) have emerged as a promising solution to capture PROs more efficiently, in less time, and without jeopardizing the validity and reliability of PRO scores [22]. The PRO, using built-in algorithms, adapts to the participant’s ability level by selecting items or a set of items from a large item bank that are sorted from the easiest to the hardest items/questions based on metrics generated from item response theory analyses. The system starts by asking a question of mid-level difficulty (eg, “Are you able to walk 1 block?”), with response options ranging from 1 (unable to do) to 5 (without any difficulty). Depending on the individual’s responses to the most recent items administered, the system selects the next item that will obtain more precise information (eg, if the answer to the mid-level item gets a high score, the next item selected has a higher level of difficulty, such as “Are you able to run to catch a bus?”). The CAT calculates a reliability estimate after each response (eg, typically 7-9 items from a larger item bank of up to 100 items) and stops asking questions when it reaches a predetermined (by the research group) reliability estimate. For this reason, it has also been called “tailored testing” that, rather than using a fixed-length questionnaire, only administers items that obtain new information. The testing software will include the text of each question as well as extensive information regarding test development and psychometric characteristics (calibration to place items on the same scale). The testing application will include item banks and computer adaptive algorithms developed and validated, providing a comprehensive PRO metric of mobility that classifies individuals with ABI across the lifespan and at all levels of mobility. Participants will be able to complete items using interactive voice recognition systems, smartphones, tablets, or desktop computers; this versatility will maximize user-centeredness and can be matched to the setting where the questionnaires are administered. The testing software will also enable researchers to compare the validity and sensitivity to detect change in mobility-related domains of CATs compared to traditional observed measures used in research and in clinical practice. Where possible and appropriate, Patient-Reported Outcomes Measurement Information System (PROMIS) CATs [23] will be used. CATs will be compared to gold standards, which include the Human Activity Profile, which focuses on low-level-to-complex mobility tasks (eg, turning in bed, walking, running), and the Assessment of Motor and Process Skills, which measures the motor and cognitive process skills of simple-to-complex community tasks (eg, retrieving a beverage from the refrigerator, shopping).

At the end of Activity 1.2, we will have developed, with our industry partner, the Participant Outcome Reporting Research System, a software application including CATs to measure low-to-high levels of mobility for persons with ABI.

##### Activity 1.3: Clinical Research System to Gather Clinician-Reported Outcomes From Clinical Research Sites

Data captured at the point of rehabilitation care (during episodes of care and as part of research trials) will provide information about the performance-based measures of mobility and rehabilitation interventions (physical, psychosocial, and cognitive). We will leverage this work to identify strategies to optimize implementation of the clinical research system (CRS), including the added value of mobility data for clinicians to identify which interventions are optimal for specific patient subgroups and to support clinical decision-making. Clinician-reported outcome (ClinRo) data obtained using performance-based evaluations of impairment, function, and mobility, as well as data on patient interventions (described in Activity 2.2), will be captured. Later, the system will be built step by step using separate modules for each type of data capture source (described in Activities 1.1-1.3) and intervention data source (eg, split-belt and conventional treadmills, virtual reality [VR], constraint, and biofeedback modules described in Activity 2.1) for patients enrolled in trials. Five years into the BRILLIANT research program, when predictive models are developed (after collecting a sufficient amount of data) and validated, the CRS will include a decision support module (see Activity 2.3) to provide recommendations for selection and implementation of appropriate mobility interventions, tailored to specific ABI characteristics (see Activity 2.2).

These ClinRo data will be captured using the CRS to be built using a commercial software package in partnership with an industry partner, allowing researchers and participants to enter data using tablets, smartphones, or computers.

##### Activity 1.4: Monitoring Data to Build a Mobility Profile

The BRILLIANT program will generate and pool biological biomedical data (BiomedO, ie, movement, forces, gaze, and neuromuscular data, portable imaging; Activity 1.1) and PRO data collected using the PRO research system (Activity 1.2) with ClinRo data using the CRS (Activity 1.3) and BrImagO data acquired using magnetic resonance imaging (MRI) during the acute, rehabilitation, and community reintegration phases. Many of the important underlying causes of aberrant motor performance have signatures that can be measured using MRI. Imaging modalities sensitive to changes in axonal structure, gray matter thickness, functional neurophysiology, neurochemical balance, and microvasculature hold the greatest promise in detecting and characterizing ABI. These imaging modalities have shown great utility for detecting and diagnosing brain damage. Ptito and coworkers [24] also successfully used MRI to evaluate brain function (blood oxygenation, flow and volume using functional magnetic resonance imaging [fMRI]) to characterize patients with mild TBI (mTBI) and also applied this to pediatric populations with Gagnon et al [24]. Importantly, the group is amongst the first to demonstrate the power of fMRI to detect and diagnose mTBI and document recovery. They also used repetitive transcranial magnetic stimulation (rTMS) to increase dorsolateral prefrontal activity and relieve symptom severity in patients with mTBI [24]. Imaging can thus be used to zoom in and resolve functional changes in frontal regions, including motor and premotor cortices.

Using individual-level data, predictive analytics will be performed to explain variability in mobility. This will enable estimation and validation of predictive models of mobility to assess the independent and joint contribution of explanatory variables, allowing us to identify mobility subgroups. Only with the data from the network of technologies, and experimentation in multiple longitudinal cohorts of persons with ABI of varying levels, and healthy controls of all ages as a comparison, will researchers be able to create such a mobility profile. The combination of the predictive models informed by expert researchers, collaborating industry and clinical partners, and end users will define the rules by which factors are encoded to develop a characteristic mobility profile.

#### Theme 2: Using the Mobility Profile for Tailored ABI Rehabilitation/Retraining

The objective of this theme is to develop cost-efficient community mobility interventions that optimize outcomes for mobility subgroups of patients with ABI. This will allow us to customize community environment mobility training by identifying, on a continuous basis, specific rehabilitation strategies and interventions that patient subgroups benefit from most.

##### Activity 2.1: Developing and Testing Enhanced Interventions for Community Mobility

Moving around in community environments requires the skills to cope with multiple simultaneous dimensions (eg, walking speed and distance, traffic level, sensory cues, postural transitions, cognitive demands). Such skills remain compromised in the majority of individuals with ABI due to nonspecific interventions [25,26]. Best-practice guidelines for all age groups recommend the use of task-specific interventions that are individually tailored, goal oriented, meaningful, engaging, progressively adapted, and of sufficient intensity and duration. As these principles are incorporated into contemporary practice, mobility interventions remain largely focused on training rhythmic/repetitive movements (eg, treadmill walking, robot-assisted movements) in clinical laboratory environments. Such practice underestimates the need for (1) movement adaptation in response to varied, meaningful real-life contexts; (2) coengagement of motor, sensory, perceptual, and cognitive systems and influence of fatigue; and (3) development of problem-solving skills/preplanned strategies [27] for community mobility. In the context of this activity, we will develop and test individually tailored interventions grounded in the best evidence in community ambulation, principles of motor learning and participatory action research with end/knowledge users.

Specifically, for patients who are in the intensive rehabilitation phase (ie, first 3 months of rehabilitation and predischarge to home/community), enhanced practice of community mobility skills (walking and wheeling) will be provided through VR applications comprising mobility tasks and environments of increasing complexity (eg, from unobstructed single-task walking a short distance to walking 100 m in a crowded mall while remembering shopping items). These VR applications will be developed collaboratively with knowledge users (patients of various ages and clinicians) as well as key industrial partners specialized in video game development and VR applications for the clinical setting. For patients with ABI in the community reintegration phase (postdischarge from intensive rehab), mobility interventions taking place within the community will be codeveloped and subsequently tested in collaboration with Cominar (owner of Alexis Nihon and partner in RehabMaLL) and other community partners (eg, ALTERGO [28]). We will implement circuit training for mobility within the rehabilitation setting to develop and test a circuit training paradigm involving complex community mobility skills in a community environment (eg, the mall). Using wearable sensor technology, we will further develop real-time biofeedback applications to provide online knowledge of performance on movement strategies (eg, lower-limb movement and weight-bearing symmetry), instantaneous walking, and propulsion speed and distance while patients move about in community environments.

##### Activity 2.2: Comparative Effectiveness of Mobility

Recent research suggests that accounting for the time spent in *specific* physical and occupational therapy activities (intervention-level data), above and beyond patients’ characteristics (patient-level data), has the potential to enhance the predictive value of models explaining rehabilitation outcomes after ABI [29]. Capturing accurate and comprehensive intervention-level data (also a ClinRo) at different times within the continuum of care (eg, acute care and then transitioning to inpatient and outpatient rehabilitation and community reintegration) allows us to identify the mediating effects of specific rehabilitation interventions to the recovery of mobility in different patient subgroups. This electronic intervention data capture application will involve validation of intervention parameters within the Canadian context (and for children/youth) and follow the most recent international reporting guidelines for interventions (eg, the template for intervention description and replication [TIDieR] checklist) [30] to create an ABI-specific treatment classification in a research-based therapeutic coding system (with a focus on mobility). This mapping application will allow adding specific treatment variables and refining the predictive power of the models to guide decision support (Activity 2.3). We will compare how the intervention mapping applies to VR, gait retraining (eg, gait circuits in a gym, split-belt treadmill, treadmill propulsion) and community-based interventions for mobility and extend to persons with ABI.

We will build on this work to create the intervention mapping module for rehabilitation in the CRS (Activity 1.3) to capture intervention parameters mentioned before and strategies used by therapists when teaching indoor/outdoor community mobility skills.

##### Activity 2.3: Mobility Decision Support Development

In this activity, we will develop and evaluate the impact of a decision support module, Rehabilitation Application-Intervention Selection Enabler (RAISE), on clinician and patient clinical decision-making in terms of mobility and practices. Using the predictive models developed in Activity 1.3 and the analysis of intervention factors developed in Activity 2.2, we will build RAISE for clinicians and conduct the first-ever implementation studies and randomized trials on the acceptability and effectiveness of RAISE for referral and discharge across transitions in care and selection of mobility retraining strategies. Since length of stay in intensive rehabilitation is shortening, therapists have to assign optimal community-based rehabilitation strategies early on in the treatment plan.

A second challenge arises when transitioning from acute care to home, when families and patients need a clear understanding of the mobility prognosis to make decisions about discharge planning resources and alterations to their home environment. RAISE will also be able to identify the best responses to questions posed by clinicians using plain language. For example, clinicians will be able to enter a question regarding the mobility prognosis, such as “When will patient X be able to climb 12 steps independently based on their present profile?” or “Will patient X be able to walk in a shopping mall independently at 6 months?” or “Would protocol A or B work best for this patient?” RAISE and natural language applications will leverage the data collected in Theme 1 and the analysis from Activities 2.1 and 2.2 to inform the intervention plan and answer these types of questions.

RAISE, as an add-on within the CRS (Activity 1.3) will be designed to allow clinicians to produce personalized intervention plans for subgroups of patients based on their mobility profile using data-driven methods.

### Ethics Approval

Ethics approval for each activity is ongoing.

## Results

### Current Status

The biomedical infrastructure and equipment have been established across the research labs and clinical sites. The participating reporting outcome system contains CAT PRO measurements (PROMs), including the Patient-Reported Outcomes Measurement Information System (PROMIS) [23] and the Assistive Technology Outcome Profile for Mobility (ATOP) [23,31], and development of other modules for feedback of PROM and mobility outcomes to participants is ongoing [32,33]; see also Alhasani et al (unpublished data, March 2021). The development of the clinical information system is also ongoing. Recruitment is expected to begin in May 2022.

### Knowledge Mobilization Process With Partners and End Users

In addition to traditional knowledge dissemination (publications in scholarly journals and conferences), the BRILLIANT team will undertake yearly interactive knowledge mobilization sessions with health system and community partners and end users. These sessions will include, but will not be limited to, dedicated work sessions with informatics specialists, patient representatives from a range of subpopulations, clinicians, and the provincial health ministry (Ministère de la Santé et des Services sociaux [MSSS]).

To ensure that experiential knowledge is effectively exchanged within the research community, the team will offer 1-day specialized training sessions. This intensive course will be directed at training clinicians, trainees, and highly qualified professionals on the use of the CRS, including the RAISE decision support system, and offered in person and virtually. Because such a commitment can be difficult for many clinicians, the BRILLIANT program will hold 4 half-day training sessions annually at clinical sites to be close to clinical programs and activities.

The mobility technologies and decision supports that identify optimal mobility interventions will have important repercussions for changes to clinical guidelines and practices. The BRILLIANT knowledge mobilization plan will also inform policies of the Quebec health care system and insurance compensation of victims of automotive accidents (eg, the Société d'Assurance Automobile du Québec [SAAQ]), workplace accidents, and violent crimes. We will further communicate our results to rehabilitation policy planners across the province and nationally to inform program planning for individuals with ABI, from rehabilitation-based interventions to community planning to support ongoing monitoring and mobility retraining support in the community. Linkages with the national and international brain injury, health professional, and accreditation associations will help to disseminate the results to persons with a vested interest in improving the lives of individuals with ABI.

Over time, through international collaborations, BRILLIANT will build partnerships to adapt and disseminate BRILLIANT innovations to other research networks and clinical settings. This work has started via the Virtual Health and Wellbeing Living Lab Infrastructure (VITALISE) initiative that brings together 3 European living labs and BRILLIANT to share resources and expertise in technology development for rehabilitation, transitions in care, and smart living environments [34-36].

Once tested and validated, we will explore commercialization opportunities with our industry partners. We will aim to make the participant reporting system, CRS, and RAISE readily available through an open source framework for other investigators to keep building and deploying RAISE, and health networks to deploy within their clinical programs.

## Discussion

### Summary

The BRILLIANT digital health and biomedical technologies will enable users to conduct research along the 2 main themes of BRILLIANT: to collect necessary data allowing the creation of a mobility profile for patients with ABI in community environments and to make use of the mobility profile for customized rehabilitation and retraining programs. The BRILLIANT research innovations will result in technology development with industry, evidence-based practices by clinical end users, and changes to clinical and governmental guidance, designed to positively impact ABI clinical outcomes.

The breakthroughs and outcomes of benefit to rehabilitation care from BRILLIANT include:

- Community-based tracking of mobility: Thousands of patients with ABI admitted annually to acute care and rehabilitation centers will see their lives improved by having a means to monitor limitations in mobility once they leave the care setting. Indeed, for children and adults who return home from acute rehabilitation, there has been limited measurement of their functioning in their own community. The community-based tracking deliverable will enable our practice teams, including physicians in the community who do not usually have access to information on functioning, to measure the mobility limitations that persons with ABI experience in their environments. Ultimately, the benefits are that patients will be able to return to their home environment sooner with greater mobility and function.
- ABI mobility limitations, interventions, and comparative effectiveness research: The patient research system to efficiently and precisely evaluate PRO domains that influence mobility, and the CRS will allow for data capture at the point of care. The CRS will include a much-needed taxonomy of rehabilitation interventions. As there is currently no rehabilitation electronic health record in the participating sites, there are no mechanisms to capture these data, limiting a clinician’s ability to evaluate patient progress, and for researchers and health administrators, to identify the care patients receive in rehabilitation. The CRS will be 1 of the first-ever rehabilitation electronic records.
- A predictive model to explain variability in mobility: The BRILLIANT data collected will allow us to continuously develop and validate predictive algorithms to explain variability in mobility. This will further enable estimation and validation of predictive models of mobility to assess the independent and joint contribution of explanatory variables, allowing us to identify mobility subgroups. For clinicians, this provides a means to identify which factors to focus on in rehabilitation mobility retraining, using a personalized approach (tailored to context) rather than the current prescriptive or recipe approach.
- Customized rehabilitation/retraining programs: The decision support tool will utilize the predictive models to build a mobility decision support, RAISE. Currently, patients receive relatively the same rehabilitation mobility training interventions that often do not target the specific factors limiting a given individual’s mobility. For patients, this means that receiving interventions that address their specific needs is likely to optimize their outcome following rehabilitation. This provides clinicians with a means to distinguish the mobility levels of patients and the interventions that are most likely to be effective.

### Conclusion

BRILLIANT represents the first large-scale use of biomedical community–based technology and health informatics to support a person-centered (personalized) approach to measuring function over time and developing interventions to improve community-based mobility. We expect the innovations created to accelerate research and discovery in ABI research and deliver the information needed for clinicians to be able to deliver cost-effective care that includes *the right intervention to the right person at the right time*, while achieving long-term functional potential and meaningful participation in the community.

## Appendix

Multimedia Appendix 1 Peer-review report by the Canada Foundation for Innovation (Multidisciplinary Assessment Committee and Special Multidisciplinary Assessment Committee).

## Abbreviations

ABI: acquired brain injury
ATOP: Assistive Technology Outcome Profile for Mobility
BRILLIANT: Biomedical Research and Informatics Living Laboratory for Innovative Advances of New Technologies in Community Mobility Rehabilitation
BrImagO: brain imaging
CAT: computerized adaptive test
CISSS: Centre intégré de santé et de services sociaux
CIUSSS: Centre intégré universitaire de santé et de services sociaux
ClinRo: clinician-reported outcome
CP: cerebral palsy
CRS: clinical research system
fMRI: functional magnetic resonance imaging
MRI: magnetic resonance imaging
mTBI: mild traumatic brain injury
PRO: patient-reported outcome
RAISE: Rehabilitation Application-Intervention Selection Enabler
RehabMaLL: Rehabilitation Living Lab in the Mall
TBI: traumatic brain injury
VR: virtual reality
