# Supplementary material for: Biomedical Research and Informatics Living Laboratory for Innovative Advances of New Technologies in Community Mobility Rehabilitation: Protocol for Evaluation and Rehabilitation of Mobility Across Continuums of Care
Source: JMIR Res Protoc. 2022 Jun 1;11(6):e12506. doi: 10.2196/12506 (PMC9201706; doi:10.2196/12506)
Supplement: Multimedia Appendix 1 [file resprot_v11i6e12506_app1.pdf]

2017 Innovation Fund
Multidisciplinary Assessment Committee (MAC) comments and Special Multidisciplinary Assessment Committee (SMAC) recommendations
Board decision made on 2017-06-20

Project leader: Ahmed, Sara

Project number: 36053

Administrative institution: McGill University

Project title: Biomedical Research and Informatics Living Lab for Innovative Advances of New Technologies (BRILLIANT) in Mobility

MAC recommendation: Full funding

Exceptional project?: No

SMAC recommendation: Conditional funding

Recommended amount: $1,959,201

Rationale for MAC recommendation:

McGill University and Université de Montréal propose to acquire essential equipment enabling both institutions to pursue their Biomedical Research and Informatics Living Laboratory for Innovative Advances of New Technologies in Community Mobility Rehabilitation (BRILLIANT-Rehab) program which was initiated in 2015. The BRILLIANT-Rehab program is the culmination of a 15-year collaboration through which one of the largest rehab research centres in Canada has been founded.

Rehabilitation is a priority area for both institutions as evidenced by an excellent track record of key investments in human capital (e.g. 30 tenure-track positions since 2002) and infrastructure (e.g. several CFI awards since 2000). The MAC noted that the investigators and their affiliated centre is globally recognised for their achievements in the transfer and application of scientific knowledge of rehabilitation as demonstrated by their record of accomplishments.

The MAC was fully supportive of the new vision to optimize the mobility of individuals with Acquired Brain Injury (ABI) by carrying out the evidence-based research program, the novelty of which lies in its large-scale use of biomedical community-based technology and health informatics to support rehabilitation strategies (physical, psychosocial, and cognitive) personalized to the individual.

The MAC agreed with the Expert Committee (EC) that the proposed objectives and activities are logically structured and feasible and overcome current lab-based rehabilitation challenges; and the integrated research deliverables demonstrate a very innovative approach. Through robust national and international linkages, the group is highly visible in the field of precision rehabilitation and is poised to assume a greater leadership role with BRILLIANT-Rehab.

The requested equipment will enable the collection of the necessary data for the creation of a mobility profile for patients with acquired brain injuries in community environments and to subsequently make use of this profile for customized rehabilitation and retraining programs. The MAC agreed with the EC that the requested infrastructure is well justified and uniquely suited to perform the proposed research; however, as a minor point, more information on the size of the patient cohorts would have better justified the number of requested portable devices. To this, the MAC remarked that the two-year data acquisition period should be extended for further impact.

Although the management plan is sound, the MAC suggested that the provisions for O&M be increased (although it did note the presence of a contingency fund and the team's high level of funding). Overall, the MAC agreed that the new infrastructure, in complementing the existing capabilities available to the team (e.g. magnetic resonance imaging systems), appears likely to form the most advanced rehabilitation program in Canada.

As ABI is a leading cause of disability in Canada, the proposal addresses issues that will affect a large number of individuals at some point in their lifetime and which represents a significant social and economic cost to society. The team has links to a large group of clinicians who both actively collaborate in rehab research and are its end-users, thus likely resulting in changes to clinical practice guidelines. Outreach to a variety of partners and other end-users through annual knowledge mobilization sessions is a noted strength of the proposal. In the context of heightened emphasis on personalized medicine, clinicians are expected to benefit from BRILLIANT-Rehab in their ability to deliver time and cost-effective care.

The research is also expected to lead to a variety of technology development opportunities with the group's industry partners (e.g. the software developed with TelASK and OMNIMED). The MAC concluded its assessment by remarking that this proposal is timely in that such predictive data tools will undoubtedly be of interest to multinationals such as Google.

Comments on partial/conditional funding:

The CFI contribution is conditional upon the institution providing Compute Canada sufficient information to enable it to determine which of its consolidated facilities is best suited to house and manage the research computing infrastructure (items #18-19). A formal confirmation must be provided by Compute Canada to the CFI.

Global Leadership	Research capacity	Benefits to Canadians
EX	SA	EX

Legend for assessment: EX = Significantly exceeds the objective, SA = Satisfies the objective, SW = Satisfies the objective with only a few minor weaknesses, PS = Partially satisfies the objective with some significant weaknesses, NS = Does not satisfy the objective due to major weaknesses

Veuillez noter que le rapport du comité d'experts pour cette proposition vous sera acheminé par le gouvernement du Québec.

Please note that the government of Québec will provide you with the expert committee report for this proposal.
